# Embryonic Stem Cells‐Derived Exosomes Endowed with Targeting Properties as Chemotherapeutics Delivery Vehicles for Glioblastoma Therapy

**DOI:** 10.1002/advs.201801899

**Published:** 2019-02-01

**Authors:** Qingwei Zhu, Xiaozheng Ling, Yunlong Yang, Juntao Zhang, Qing Li, Xin Niu, Guowen Hu, Bi Chen, Haiyan Li, Yang Wang, Zhifeng Deng

**Affiliations:** ^1^ Department of Neurosurgery Shanghai Jiaotong University Affiliated Sixth People' Hospital No. 600 Yishan Road Shanghai 200233 China; ^2^ Institute of Microsurgery on Extremities Shanghai Jiaotong University Affiliated Sixth People' Hospital No. 600 Yishan Road Shanghai 200233 China; ^3^ Med‐X Research Institute, School of Biomedical Engineering Shanghai Jiao Tong University 1954 Huashan Road Shanghai 200030 China

**Keywords:** blood–brain barrier, drug delivery system, exosomes, glioblastoma, human embryonic stem cells

## Abstract

Exosomes are nanosized membrane vesicles (30–100 nm) that can easily penetrate the blood–brain barrier, safely deliver therapeutic drugs, and be modified with target ligands. Embryonic stem cells (ESCs) provide abundant exosome sources for clinical application due to their almost unlimited self‐renewal. Previous studies show that exosomes secreted by ESCs (ESC‐exos) have antitumor properties. However, it is not known whether ESC‐exos inhibit glioblastoma (GBM) growth. In this study, the anti‐GBM effect of ESC‐exos is confirmed and then c(RGDyK)‐modified and paclitaxel (PTX)‐loaded ESC‐exos, named cRGD‐Exo‐PTX are prepared. It is then investigated whether the engineered exosomes deliver more efficiently to GBM cells versus free drug alone and drug‐loaded ESC‐exos using an in vitro GBM model and in vivo subcutaneous and orthotopic xenografts model. The results show that cRGD‐Exo‐PTX significantly improves the curative effects of PTX in GBM via enhanced targeting. These data indicate that ESC‐exos are potentially powerful therapeutic carriers for GBM and could have utility in many other diseases.

## 1 Introduction

Glioblastoma (GBM) is the most common primary malignant tumor of central nervous system with high mortality and disability rate.^1^, ^2^ Despite the high drug resistance of GBM, another pivotal reason for this poor prognosis is that blood–brain barrier (BBB) limits the efficacy of chemotherapy drugs to reach the tumor region.^3^, ^4^, ^5^ Hence, developing well‐designed drug delivery strategy to overcome BBB is an urgent demand in achieving efficient therapy of GBM.

In previous studies, significant efforts have been made to develop synthetic drug delivery systems to penetrate the biological barriers.^6^, ^7^ However, these synthetic nanocarriers still have some problems, such as biotoxicity and inefficient BBB penetrating ability.^8^, ^9^ Exosomes are a class of lipid bilayer extracellular vesicles that are endogenously secreted by almost all types of mammalian cells, with a diameter distribution of 30–100 nm and respond for cell–cell communication through transporting molecules from parent cells to recipient cells.^10^, ^11^, ^12^ In the view of naturally derived nanocarriers, exosomes have been recognized as excellent nanovehicles for drug delivery due to their low biotoxicity and diverse biofunctions such as immune privilege, cargo protection, and most importantly stringent biological barriers penetrating ability.^13^, ^14^, ^15^, ^16^, ^17^ It has been reported that exosomes exhibit intrinsic tissue or cell‐targeting properties owing to their particular surface structures like tetraspanins and integrins.^18^, ^19^ Furthermore, better target ability of exosomes can be acquired via engineering surface molecules.^20^, ^21^, ^22^ Despite advances in exosome‐based drug carriers, a suitable cell type that is ideal for exosome derivation should be identified.

Human embryonic stem cells (ESCs) are pluripotent stem cells derived from the inner cell mass of blastocysts and can be maintained virtually and indefinitely undifferentiated in vitro culture.^23^, ^24^ Furthermore, they also have the ability of infinite proliferation.^25^ Therefore, ESCs can produce exosomes (ESC‐exos) with stable properties in large scale, which are very important factors for exosomes' clinical application. In addition, ESC microenvironment has been confirmed to suppress oncogenic phenotypes of carcinoma cells. Pioneer works indicated that malignant cancer cells were reprogrammed to a more differentiated, less aggressive phenotype in embryonic microenvironment.^26^, ^27^, ^28^, ^29^ ESC‐exos harbor substances that mirror the content of the embryonic microenvironment, which could be the main effectors of the ESCs‐mediated antitumor effect. Moreover, Zhou et al. found that ESC‐exos could reprogram human mammary carcinoma cells toward a less malignant cell phenotype.^30^ Thus, comparing with other cell types, ESCs are very suitable producers of exosomes to develop exosome‐based chemotherapeutic carriers in cancer therapy.

However, although exosomes show the ability to penetrate BBB, many researches show that intravenously injected exosomes are manly distributed in liver or spleen, and very small partial of injected exosomes retain in brain or in tumor site.^31^, ^32^ Thus, before utilizing ESC‐exos for glioma therapy, efficient strategy should be taken to solve the problem. Direct modification of exosomes surface with tumor‐targeting ligand has been proved to be efficient in enhancing tumor retention of exosomes.^21^, ^33^ Cyclo (Arg‐Gly‐Asp‐D‐Tyr‐Lys) peptide (c(RGDyK)) is a well‐known targeting ligand for cancer chemotherapy and exhibits high affinity with α_v_β_3_ integrin receptors that over‐express on the surface of actively proliferating endothelium of tumor tissues such as GBM,^21^, ^34^ prostate cancer,^35^ and lung cancer.^36^ These studies show that nanocarriers modified with c(RGDyK) can more efficiently deliver chemotherapeutic agents into cancer cells and could significantly improve antitumor effect. In addition, exosomes modified with c(RGDyK) have the strong ability to cross the BBB.^21^, ^37^ However, whether ESC‐exos modified with c(RGDyK) can enhance ESC‐exos distribution in glioma site and lead to more efficient therapy for glioma is stillunknown.

Based on the above statement, in this study, we developed a tumor‐targeting ESC‐exos (cRGD‐ESC‐exos) delivery system by modifying ESC‐exos with c(RGDyk) peptide for glioma therapy. We expect that through combining advanced features of ESC‐exos and targeting ligand, an efficient exosomes‐based strategy can be developed for glioma therapy, and our work can also promote the application of ESC‐exos for drug delivery.

## 2 Results and Discussion

### 2.1 Characterization of ESCs and ESC‐Exos

**Figure 1:**
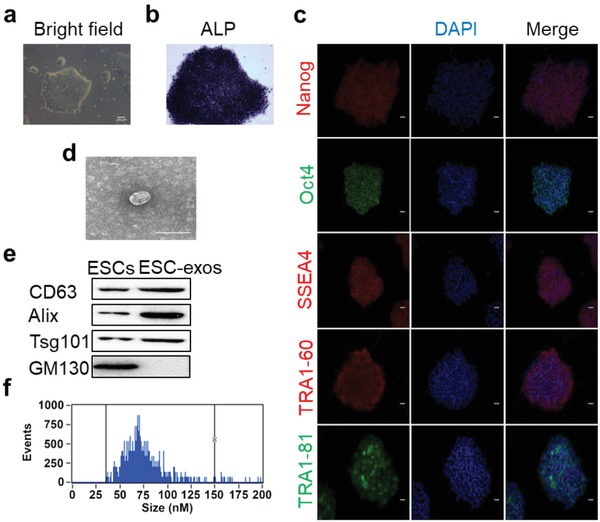
Characterization of ESCs and ESC‐exos. a) Morphology and b) ALP activity of ESCs. Scale bar, 200 µm. c) Immunofluorescence staining of ESCs markers Nanog (red), Oct4 (green), SSEA4 (red), TRA1‐60 (red), and TRA1‐81 (green) with a nucleus marker DAPI (blue). Scale bar, 50 µm. d) Transmission electron micrograph of ESC‐exos. Scale bar, 100 nm. e) Western blotting analysis of CD63, Alix, Tsg101, and GM130 from ESCs and ESC‐exos. f) Flow nanoanalyzer analysis of particle size distribution of ESC‐exos, and the average diameter of ESC‐exos was 70 ± 18 nm.

ESCs cultured on Matrigel formed a compact colony with sharp border (**Figure** **1**a). Immunostaining analysis showed that all the cells in a sphere expressed alkaline phosphatase (ALP, Figure 1b) and pluripotency marker molecules, including Nanog, Oct4, SSEA4, TRA1‐60, and TRA1‐81 (Figure 1c). These features of the cultured cells are in conformity with the characteristics of ESCs.^38^ Thereafter, ESC‐exos were purified from cell culture supernatant and characterized by transmission electron microscopy (TEM), Western blotting, and flow nanoanalyzer (NanoFCM). TEM image clearly revealed that ESC‐exos exhibited a round‐shaped morphology with a diameter of about 75 nm (Figure 1d). Western blot confirmed that ESC‐exos expressed exosomal specific markers CD63, Alix, and TSG101, but not the negative marker Golgi membrane bound protein GM130 (Figure 1e). Flow nanoanalyzer analysis showed that the average diameter is 70.2 ± 18 nm (Figure 1f) and the concentration of the ESC‐exos was approximately 1 × 10^11^ particles mL^−1^. These data demonstrate that we have successfully extracted and purified ESC‐exos from ESCs culture medium.

### 2.2 ESC‐Exos Display GBM Suppressive Activity Both In Vitro and In Vivo

**Figure 2:**
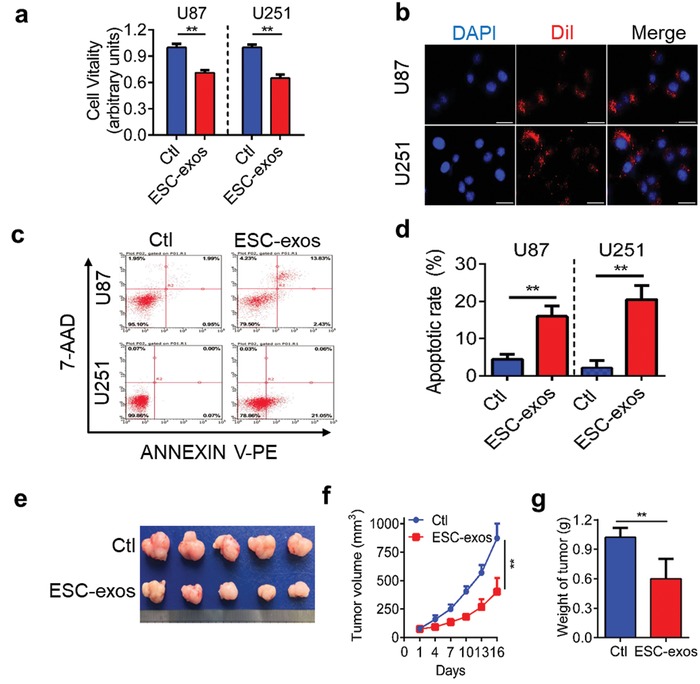
ESC‐exos display GBM suppressive activity in vitro and in vivo. a) Antiproliferative activity of ESC‐exos against U87 and U251 cells. Cells were treated with PBS (Ctl) or ESC‐exos (1 × 10^9^ particles mL^−1^) for 48 h. The cell vitality was measured by the CCK‐8 assay. b) Representative images of uptake of ESC‐exos by U87 and U251 cells in vitro. Nuclei were stained with DAPI (blue). Scale bar, 25 µm. c) U87 and U251 cells were treated with PBS (Ctl) or ESC‐exos (1 × 10^9^ particles mL^−1^) for 48 h, stained by Guava Nexin reagent or propidium iodide and then analyzed by flow cytometry. d) Quantitative analysis of cell apoptotic rate in each group. e) Representative images of subcutaneous GBM tumors. f) Tumor growth curves and g) tumor weight at the indicated days. *p < 0.05, **p < 0.01.

To confirm whether ESC‐exos have a broad spectrum of anticancer activities and especially for GBM, we cultured six types of cancer cell lines, including human GBM cell lines (U87 and U251), lung cancer cell line (A549), hepatocellular carcinoma (HCC) cell line (HepG2), melanoma cell line (B16), breast cancer cell line (MDA‐MB‐231), and prostatic cancer cell line (DU145), with ESC‐exos (1 × 10^9^ particles mL^−1^) containing medium for 48 h. As shown in **Figure** **2**a and Figure S1 in the Supporting Information, cell viability assay (CCK‐8 assay) revealed that ESC‐exos significantly decreased the viability of these cancer cells. To further confirm the effect of ESC‐exos in GBM cells, U87 and U251 cell lines were used to undertake follow‐up experiments.

In order to exert their effects on U87 and U251 cells, ESC‐exos need to be up‐taken by these cells. Thus, ESC‐exos were labeled with lipophilic fluorescent dye DiI and added to U87 and U251 cell culture mediums. After 12 h incubation, ESC‐exos were efficiently internalized by U87 and U251 (Figure 2b). To evaluate the effects of cell apoptosis, flow cytometry analyses using Annexin V‐PE/7‐AAD labeling were preformed to detect the induction of cell apoptosis following ESC‐exos (1 × 10^9^ particles mL^−1^) treatment. The percentage of apoptotic cells significantly increased in ESC‐exos group, compared with control group (Figure 2c,d). These data indicate that ESC‐exos affect GBM cell growth by inhibiting cell viability and promoting cell apoptosis in vitro.

To evaluate whether ESC‐exos are able to affect the GBM growth in vivo, we performed athymic nude mice bearing subcutaneous U87 GBM xenografts. Once tumor masses grew to about 200 mm^3^ (≈2 weeks), mice were intravenous injected with either phosphate buffered saline (PBS, control group, Ctl, 125 µL) or ESC‐exos (125 µL, 1 × 10^11^ particles mL^−1^) every other day for 2 weeks. Tumor bodies removed from these mice are shown in Figure 2e, and tumor growth curves and tumor weight are shown in Figure 2f,g. The size of tumors in mice injected with ESC‐exos (401.3 ± 122.3 mm^3^) was significantly smaller than tumors in PBS‐injected mice (872.4 ± 128.2 mm^3^; *p* <0.01) by the end of the study. Tumors in ESC‐exos‐treated group were also markedly decreased in weight compared to tumors in PBS‐treated group (1.0 ± 0.1 g vs 0.6 ± 0.2 g; *p* < 0.05). These data show that the tumor growth was significantly suppressed in the presence of ESC‐exos compared with PBS group. Collectively, these data demonstrate that ESC‐exos exhibit anti‐GBM effect in vivo.

Previous studies have documented the tumor suppressive effects of ESCs microenvironment.^26^, ^27^, ^28^, ^29^ ESCs microenvironment can reprogram aggressive cancer cells toward a benign phenotype by diminished clonogenicity and tumorigenicity. Nodal signaling pathway has been shown to be involved in such process.^39^, ^40^ ESC‐exos harbor substances that mirror the content of their cell of origin and have been reported to reprogram mammary carcinoma cells to a benign stage and reduce their tumorigenicity.^30^ The proposed mechanism by which ESC‐exos reprogram malignant cancer cells was related to transferring their cargo to target cancer cells.^30^, ^41^ This study applied in vitro and in vivo models to specifically demonstrate the anti‐GBM properties of ESC‐exos. However, the mechanisms underlying this phenomenon were not illusive in this study. Based on pioneer reports, the possible underlying mechanisms may be that ESC‐exos contain ESC‐specific reprogramming factors that can be delivered to target GBM cells, which subsequently revert them to a benign phenotype.

### 2.3 Preparation of cRGD‐Exo‐PTX

The poor ability of many drugs to cross BBB greatly limits the efficacy of chemotherapies for GBM. Exosomes possess intrinsic ability to cross BBB and hence, suitable to overcome the problems associated with powerful and potential drugs that cannot reach to clinical trials because of their BBB impermeability.^42^, ^43^ ESCs can provide abundant exosomes sources for clinical application and ESC‐exos have notable anti‐GBM effects as we have been proved above. These characteristics make ESC‐exos a promising drug delivery carrier for GBM therapy. However, the biodistribution studies of unmodified exosomes after intravenous injection revealed a rapid accumulation of exosomes in organs of liver and spleen, and few exosomes were delivered to the brain. Thus, targeting characteristics of ESC‐exos require improvement before they can be used to deliver therapies against GBM. It has been well documented that c(RGDyK) peptides conjugated drug delivery systems aiming at α_v_β_3_ integrin for active tumor‐targeting therapy.^44^ Paclitaxel (PTX) is a mitotic inhibitor to tumor cells and widely used for the treatment of solid tumors. Although, PTX shows effective anti‐GBM activity, it is not able to produce a therapeutic effect in vivo because of its low BBB permeability.^45^, ^46^

**Figure 3:**
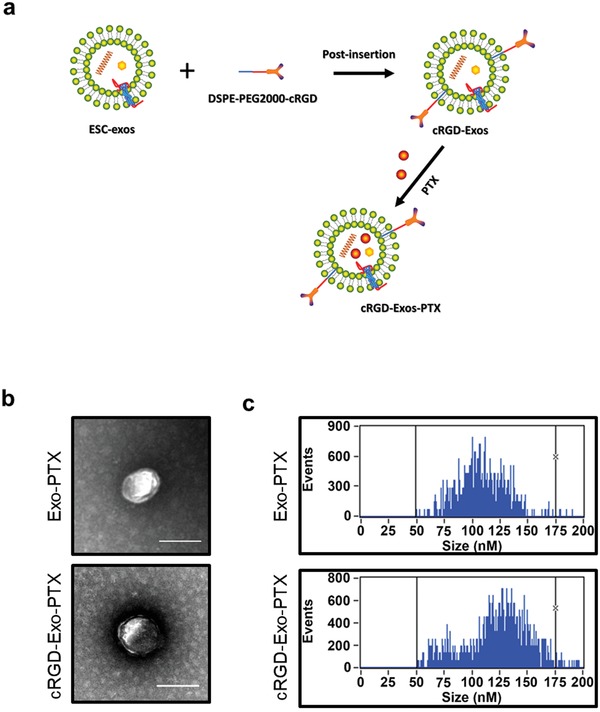
Tumor‐targeting modification and drug encapsulation of ESC‐exos. a) Schematic diagram of conjugating c(RGDyK) peptide and PTX loading into ESC‐exos. b) Representative transmission electron micrograph of Exo‐PTX and the cRGD‐Exo‐PTX. Scale bar, 100 nm. c) The particle size distribution of Exo‐PTX and the cRGD‐Exo‐PTX analyzed by flow nanoanalyzer. The average diameter of Exo‐PTX was 107 ± 20 nm, and the average diameter of cRGD‐Exo‐PTX was 125 ± 27 nm.

To obtain the suitable engineered ESC‐exos for the following experiments, ESC‐exos were first conjugated with c(RGDyK) by postinsertion method, and then loaded with PTX by coincubation, named cRGD‐Exo‐PTX (**Figure** **3**a). The morphology of Exo‐PTX (ESC‐exos loaded with PTX) and cRGD‐Exo‐PTX were assessed by TEM, and they both showed round spherical vesicles (Figure 3b). The distribution range of particle diameter was analyzed by NanoFCM. The average diameter of Exo‐PTX and cRGD‐Exo‐PTX is 107 ± 20 and 125.2 ± 27 nm, respectively (Figure 3c). These results indicate that cRGD‐Exo‐PTX and Exo‐PTX maintained the integrity after cRGD modification and drug loading.

### 2.4 Targeting Capability of cRGD‐Exo‐PTX In Vitro and In Vivo

**Figure 4:**
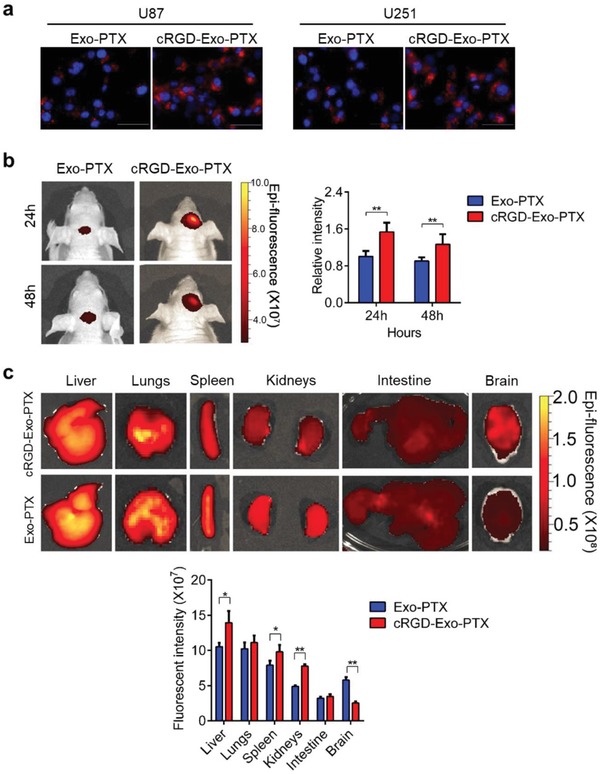
Targeting capability of cRGD‐Exo‐PTX in vitro and in vivo. a) Representative fluorescence images of in vitro internalization of DiI‐labeled Exo‐PTX and the cRGD‐Exo‐PTX after 12 h incubation with U87 and U251 cells. Scale bar, 25 µm. b) The in vivo fluorescence images of Exo‐PTX and the cRGD‐Exo‐PTX at 24 and 48 h after intravenous injection, and quantitation of fluorescence intensity by the Spectrum/CT software (*n =* 3) . c) The ex vivo fluorescence pictures of different organs were captured at 24 h after ESC‐exos and cRGD‐Exo‐PTX injection, and the quantitation of fluorescence intensity (*n =* 3) . *p < 0.05, **p < 0.01.

After preparation of cRGD‐Exo‐PTX, we assessed the targeting ability of cRGD‐Exo‐PTX. U87 and U251 cells were incubated with DiI‐labeled Exo‐PTX and cRGD‐Exo‐PTX, and then the fluorescence signal was observed with fluorescence microscope. The concentration of Exo‐PTX and the cRGD‐Exo‐PTX was 1 × 10^9^ particles mL^−1^. The stronger signal was shown in U87 and U251 cells incubated with cRGD‐Exo‐PTX than that of cells incubated with Exo‐PTX (**Figure** **4**a), which suggests that cRGD‐Exo‐PTX had significant GBM cell‐targeting ability in vitro. Furthermore, we confirmed the accumulation ability of cRGD‐Exo‐PTX in vivo using orthotopic xenografts. Exo‐PTX and cRGD‐Exo‐PTX were labeled with DiR, respectively. Then, 125 µL DiR‐labeled Exo‐PTX or cRGD‐ Exo‐PTX (1 × 10^11^ particles mL^−1^) was intravenously injected into tumor‐bearing mice 1 week after orthotopic implantation (*n* = 3). The intensity of fluorescence signal was recorded at 24 and 48 h by live imaging systems. As shown in Figure 4b, the fluorescence intensity in the tumor region of cRGD‐Exo‐PTX‐treated group was significantly higher than Exo‐PTX‐treated group. The major organs (brain, liver, spleen, lungs, kidneys, and intestine) were then collected, and the fluorescence signal in these organs was further detected. As shown in Figure 4c, the fluorescence in the brain was weaker in the Exo‐PTX group than that in cRGD‐Exo‐PTX group. Conversely, the fluorescence in other organs (liver, spleen, lungs, kidneys, and intestine) was significantly stronger in Exo‐PTX‐treated mice than that in cRGD‐Exo‐PTX‐treated mice. These results suggested that cRGD‐Exo‐PTX could accumulate in glioma site more efficiently than Exo‐PTX, demonstrating the tumor‐targeting ability of cRGD‐Exo‐PTX.

### 2.5 Targeted Therapy of cRGD‐Exo‐PTX In Vitro

**Figure 5:**
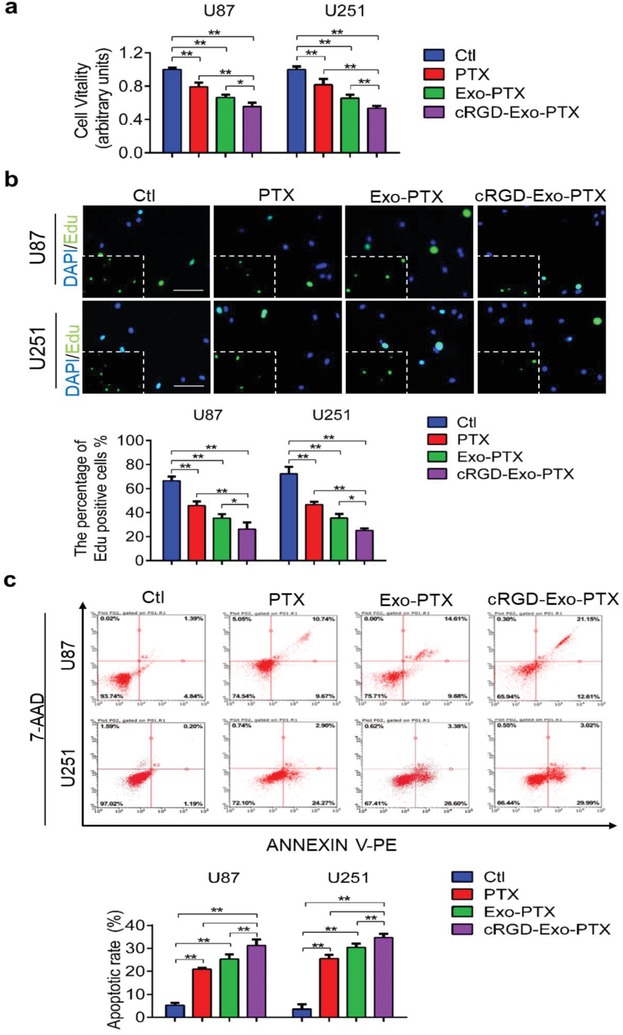
Targeted therapy of cRGD‐Exo‐PTX in vitro. a) Cell viability was evaluated using the CCK‐8 assay. b) Cell proliferation ability was assessed by the EdU incorporation assay. c) Cell apoptotic rate was detected using flow cytometry assay. *p < 0.05, **p < 0.01.

After validating the targeting ability of cRGD‐Exo‐PTX, we assessed the antiproliferation effects of cRGD‐Exo‐PTX on U87 and U251 cells by CCk8 and Click‐iT EdU assay. U87 and U251 cells were incubated with PBS (control group, Ctl), PTX (3.2 µg mL^−1^), Exo‐PTX (1 × 10^9^ particles mL^−1^), and cRGD‐Exo‐PTX (1 × 10^9^ particles mL^−1^) for 48 h. The results showed that cRGD‐Exo‐PTX could more effectively reduce cell viability of U87 and U251 cells than PBS, free PTX, and Exo‐PTX (**Figure** **5**a,b). Then, we assessed cell apoptotic rate in these cultures. With cRGD‐Exo‐PTX treatment, there was a significant increase in the percentage of apoptosis cells in U87 and U251 compared to those treated with PBS, PTX, and Exo‐PTX (Figure 5c). These results indicate that cRGD‐Exo‐PTX exert stronger inhibitory effect than free PTX and Exo‐PTX in U87 and U251 cells, which confirmed the high antitumor efficacy of cRGD‐Exo‐PTX in vitro.

### 2.6 Targeted Therapy of cRGD‐Exo‐PTX In Vivo

**Figure 6:**
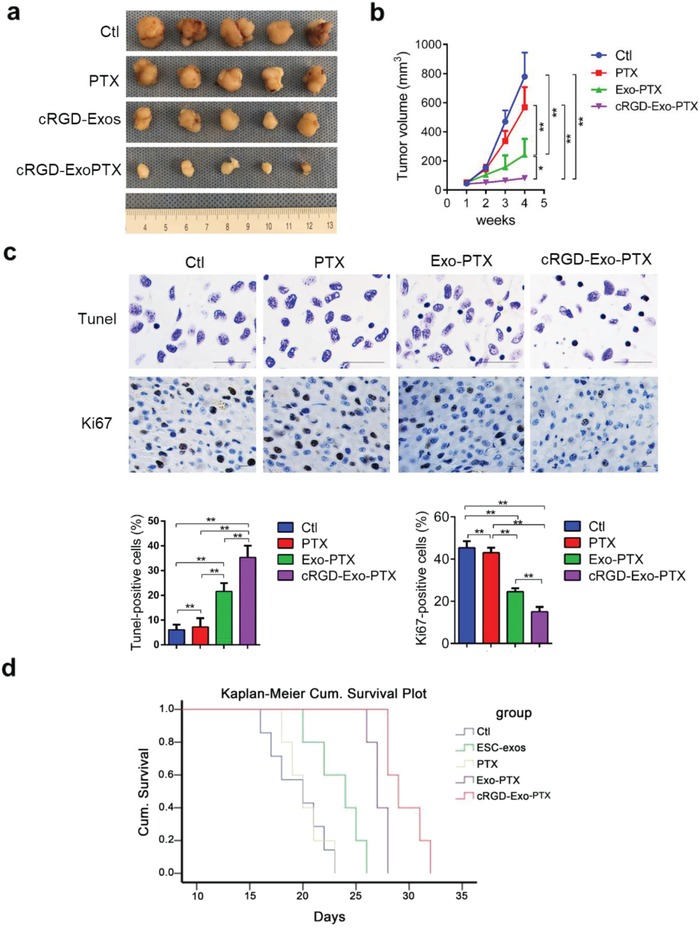
Targeted therapy of cRGD‐Exo‐PTX in vivo. a) Representative images of subcutaneous U87 GBM tumors harvested from each group treated with PBS (Ctl group), PTX, Exo‐PTX, and cRGD‐Exo‐PTX. b) Volumetric measurement of subcutaneous xenograft tumors in each group at the indicated time. c) Representative images of immunohistochemistry analysis to detect TUNEL and Ki‐67 expression in tumors originated from different agent‐treated group. d) The survival time in mice bearing orthotopic brain tumor treated with PBS, ESC‐exos, PTX, Exo‐PTX, and cRGD‐Exo‐PTX. *p < 0.05, **p < 0.01.

Encouraged by these results, we further observed the therapeutic effect of cRGD‐Exo‐PTX on subcutaneous tumor‐bearing mice. Mice was treated via caudal vein every other day with PBS (control group, Ctl, 125 µL), PTX (320 µg mL^−1^; 125 µL), Exo‐PTX (1 × 10^11^ particles mL^−1^; 125 µL), and cRGD‐Exo‐PTX (1 × 10^11^ particles mL^−1^; 125 µL) for 2 weeks. As shown in **Figure** **6**a,b, PTX, Exo‐PTX, and cRGD‐Exo‐PTX treatment significantly decreased the tumor volume when compared with PBS‐treated group by the end of the study (778.7 ± 165.4 mm^3^ in PBS group vs 567.9 ± 138.9 mm^3^ in PTX group, *p* < 0.05; vs 242.2 ± 109.0 mm^3^ in Exo‐PTX group, *p* < 0.01; vs 81.2 ± 14.0 mm^3^ in cRGD‐Exo‐PTX group). Compared with the free PTX and Exo‐PTX group, the tumor growth was significantly suppressed in the cRGD‐Exo‐PTX group (81.2 ± 14.0 mm^3^ in cRGD‐Exo‐PTX group vs 567.9 ± 138.9 mm^3^ in PTX group, *p* < 0.01, vs 242.2 ± 109.0 mm^3^ in Exo‐PTX group, *p* < 0.05). The body weight showed no significant difference in each group (Figure S2, Supporting Information). Furthermore, the number of Ki67 positive cells was significantly decreased and the number of terminal deoxynucleotidyl transferase dUTP nick end labeling (TUNEL) positive cells was notably increased after treatment with cRGD‐Exo‐PTX compared with those of in PBS, PTX, and Exo‐PTX groups (Figure 6c).

To evaluate the long‐term effect of the cRGD‐Exo‐PTX, overall survival was recorded in orthotopic brain tumor model. Mice was treated via caudal vein every other day with PBS (control group, Ctl, 125 µL), ESC‐exos(1 × 10^11^ particles mL^−1^; 125 µL), PTX (320 µg mL^−1^; 125 µL), Exo‐PTX (1 × 10^11^ particles mL^−1^; 125 µL), and cRGD‐Exo‐PTX (1 × 10^11^ particles mL^−1^; 125 µL) every other day. The Kaplan–Meier survival curves indicated that the mean survival in orthotopic brain tumor‐bearing mice treated with cRGD‐Exo‐PTX was much longer than other groups (Figure 6d). These results suggest that cRGD‐Exo‐PTX has stronger targeted therapy than ESC‐exos, PTX, and Exo‐PTX in vivo.

## 3 Conclusion

In summary, we confirmed the GBM inhibitory effect of ESC‐exos in vitro and in vivo. Then ESC‐exos were conjugated with tumor‐targeted c(RGDyK) peptide and loaded with PTX synchronously. The engineered exosomes, named cRGD‐Exo‐PTX, were demonstrated to possess excellent GBM‐targeting ability and improve the curative efficacy of PTX in GBM models. Collectively, our research designs a new type of GBM‐targeting exosomes, and provides a powerful tool for brain tumor therapy.

## 4 Experimental Section

*Cell Culture*: ESCs (H9; provided by the Institute of Biochemistry and Cell Biology of Chinese Academy of Sciences) were routinely cultured in mTeSR1 media (Stem Cell Technologies, Vancouver, Canada) on Matrigel‐coated plates and passaged every 4–5 days. The human GBM cell lines U87 and U251, lung cancer cell line A549, HCC cell line HepG2, melanoma cell line B16, breast cancer cell line MDA‐MB‐231, and prostatic cancer cell line DU145 were purchased from the Cell Bank of the Chinese Academy of Sciences (Shanghai, China) and propagated in Dulbecco's modified Eagle medium containing 10% fetal bovine serum (Gibco Grand Island, USA). All cell lines were incubated at 37 °C in humidified atmosphere of 5% CO_2_.

*Isolation and Purification of ESC‐Exos*: ESC‐exos were purified from cell culture supernatant as previously described.^47^, ^48^ ESC culture medium was collected with a pipette when the cells were 80–90% confluent. The supernatant was first centrifuged at 300 × *g* for 10 min and 2000 × *g* for 10 min and then filtered through a 0.22 µm sterilized filter membrane (Steritop, Millipore) to remove cells and cell debris. The filtrate was pelleted by ultracentrifugation (Beckman ultracentrifuge, Beckman Coulter) at 1 00 000 × *g* for 70 min. The pelleted exosomes were washed twice in PBS, resuspended in PBS, and stored at −80 °C until used.

*Characterization of ESC‐Exos*: Exosomal markers CD63 (1:1000; Abcam, Cambridge, UK), Alix (1:1000; Abcam), and TSG101 (1:1000; Santa Cruz, Dallas, USA) were determined by using Western blot. The morphology and size distribution of exosomes were identified by using TEM (Hitachi, Tokyo, Japan) and flow nanoanalyzer (Xiamen, China) instruments.

*Preparation of Tumor‐Targeted Exosomes and Drug Encapsulation*: c(RGDyK) was incorporated into ESC‐exos by postinsertion method according to previous work.^49^ Briefly, 1,2‐distearoyl‐sn‐glycero‐3‐phosphoethanolamine‐*N*‐[methoxy(polyethylene glycol)‐2000]‐c(RGDyK) (DSPE‐PEG2000‐cRGDyK, Ruixi, Xi'an, China) was dissolved in 4‐(2‐hydroxyethyl)‐1‐piperazineethanesulfonic acid buffer for 15 min at 60 °C to form micelles. Then, the ESC‐exos suspension was mixed with the above suspension for 2 h at 40 °C. After cooling to room temperature, exosomes were immediately purified by size‐exclusion chromatography to get cRGD‐modified exosomes (cRGD‐exos). After that, PTX was loaded into exosomes by co‐incubation. Briefly, PTX was dissolved in dimethyl sulfoxide with 1 mg mL^−1^ concentration. Then, the above solution was mixed with exosomes or cRGD‐exos suspension (1 × 10^10^ mL^−1^). The mixture was incubated at room for 2 h. Finally, PTX‐loaded exosomes were purified by ultracentrifugation. The PTX‐loading content was detected by high performance liquid chromatography, which was 32 µg per 10^10^ particles.

*Internalization of ESC‐Exos In Vitro*: Exosomes were labeled with the Molecular Probes' Vybrant 1,1′‐dioctadecyl‐3,3,3′,3′‐tetramethylindocarbocyanine perchlorate (DiI, ThermoFisher Scientific) according to the manufacturer's instructions. U87 and U251 cells were exposed to the labeled exosomes (1 × 10^9^ particles mL^−1^) for 12 h. For fluorescence microscopy, the cells were fixed with 4% paraformaldehyde for 20 min, permeabilized in 0.3% Triton‐X 100 in PBS for 5 min, and stained nucleic acid with 4′,6‐diamidino‐2‐phenylindole (DAPI, Beyotime Biotechnology, China). The results were examined with fluorescence microscope (Leica Microsystems, Wetzlar, Germany).

*Cell Proliferation Assays*: The cell viability of tumor cells was evaluated by Cell Counting Kit‐8 (CCK‐8; Dojindo, Japan) assay and Click‐iT EdU assay according to the protocol described.

*Cell Apoptosis Assay*: After treatment, single‐cell suspensions were washed with PBS, stained with Guava Nexin reagent which contained Annexin V‐PE and 7‐AAD for 20 min at room temperature in the dark and then analyzed by a Guava easyCyte flow cytometer.

*Western Blotting Analysis*: Total protein was extracted from tissues and cells by using radio immunoprecipitation assay lysis buffer. Equal amounts of protein were separated on 10% sodium dodecyl sulfate polyacrylamide gel electrophoresis gel and then transferred onto polyvinylidene fluoride membranes. After blockade with 5% skim milk for 1 h, the membranes were incubated with primary and the horseradish peroxidase‐conjugated secondary antibodies and detected by using ECL detection system (Amersham Pharmacia Biotech, Little Chalfont, UK).

*Establishment of Subcutaneous Xenograft and Orthotopic Glioma‐Bearing Nude Mouse Model*: All animal experimental protocols were approved by the Animal Research Committee of Shanghai Jiao Tong University Affiliated Sixth People's Hospital (SYXK (Shanghai, China) 2011‐0128, January 1, 2011). Female athymic nude mice aged 5–6 weeks were purchased from SLAC Laboratory Animal Company (Shanghai, China). GBM xenografts were produced by subcutaneously injecting U87 cells (2 × 10^6^ cells mL^−1^) in the right flank of the mice. The length (*L*) and width (*W*) of the subcutaneous tumors were measured and calculated the tumor volumes by the formula (*L* × *W* ^2^)/2. The body weight was also measured in each group . Mice were euthanized on the indicated time, and the tumor tissues were acquired, weighed, and used for the next immunohistochemical staining. The orthotopic GBM mouse model was first established by stereotaxic injection of U87 cell suspensions, 1 week later, the mice were treated via caudal vein with PBS, ESC‐exos, PTX, Exo‐PTX, and cRGD‐Exo‐PTX every other day. The concentration of exosomes was 1 × 10^11^ particles mL^−1^. The amount of PTX in the cRGD‐Exo‐PTX group was 320 ug mL^−1^, which is the same as the PTX alone group, and the volume of each injection was 125 µL. The survival time was recorded in each group.

*In Vivo Biodistribution of Exosomes*: Exosomes were labeled with the Molecular Probes' Vybrant (ThermoFisher Scientific)1,1′‐dioctadecyl‐3,3,3′,3′‐tetramethylindotricarbocyanine iodide (DiR) according to the manufacturer's instructions and the labeled exosomes were washed three times with PBS and concentrated with Amicon centrifugal filter devices (Millipore). Nude mice bearing orthotopic GBM were used to detect the biodistribution of exosomes. Mice (*n* = 3) were intravenously administered with 125 µL near‐infrared fluorescent dye DiR‐labeled ESC‐exos, and cRGD‐Exo‐PTX (1 × 10^11^ particles mL^−1^). The fluorescence images of mouse body were then recorded by the IVIS Spectrum/CT imaging system (Perkin‐Elmer, USA) at 24 and 48 h after the injection.

*Histopathological Examination*: For immunohistochemical staining, the formalin‐fixed tumor tissues were cut into sections (4 µm thickness) for Ki67 and TUNEL staining.

*Statistical Analysis*: Each experiment was repeated at least three times. All data were plotted as the mean ± SD, and the SPSS 19.0 software (SPSS Inc., Chicago, IL, USA) was used for data analysis. A comparison of the two groups was performed by Student's *t*‐test. One‐way Analysis of Variance with Bonferroni post hoc Tukey test was applied for comparison among multiple groups. Survival analysis was performed using the Kaplan–Meier method and compared by the log‐rank test. *p*‐Values less than 0.05 were considered to indicate statistically significant.

## Conflict of Interest

The authors declare no conflict of interest.

## Supporting information

Supplementary
