# Supplementary material for: Embryonic Stem Cells‐Derived Exosomes Endowed with Targeting Properties as Chemotherapeutics Delivery Vehicles for Glioblastoma Therapy
Source: Adv Sci (Weinh). 2019 Feb 1;6(6):1801899. doi: 10.1002/advs.201801899 (PMC6425428; doi:10.1002/advs.201801899)
Supplement: Supplementary file 1 — Supplementary [file ADVS-6-1801899-s001.pdf]

Copyright WILEY-VCH Verlag GmbH & Co. KGaA, 69469 Weinheim, Germany, 2019.

ADVANCED SCIENCE
Open Access

Supporting Information

for *Adv. Sci.,* DOI: 10.1002/advs.201801899

Embryonic Stem Cells-Derived Exosomes Endowed with Targeting Properties as Chemotherapeutics Delivery Vehicles for Glioblastoma Therapy

Qingwei Zhu, Xiaozheng Ling, Yunlong Yang, Juntao Zhang, Qing Li, Xin Niu, Guowen Hu, Bi Chen, Haiyan Li, Yang Wang, and Zhifeng Deng**

Supporting Information

Embryonic Stem Cells-derived Exosomes Endowed with Targeting Properties as Chemotherapeutics Delivery Vehicles for Glioblastoma Therapy

Qingwei Zhu[1,#], Xiaozheng Ling[1，#], Yunlong Yang[2，#], Juntao Zhang[2] , Qing Li[2], Xin Niu[2],Guowen Hu[1], Bi Chen[2], Haiyan Li, Yang Wang[2,*] , Zhifeng Deng[1,*]

1. Department of Neurosurgery, Shanghai Jiaotong University Affiliated Sixth People' Hospital, No.600 Yishan Road, Shanghai 200233, China
2. Institute of Microsurgery on Extremities, Shanghai Jiaotong University Affiliated Sixth People' Hospital, No.600 Yishan Road, Shanghai 200233, China
3. Med-X Research Institute, School of Biomedical Engineering, Shanghai Jiao Tong University, 1954 Huashan Road, Shanghai 200030, China

***Corresponding Author:**

Prof. Zhi-Feng Deng

Address: Department of Neurosurgery, Shanghai Jiao Tong University Affiliated Sixth People's Hospital, No. 600 Yishan Road, Shanghai

200233 (China).

Tel. +86-21-64701361, E-Mail: dengzf63@126.com

Prof. Yang Wang

Address: Institute of Microsurgery on Extremities, Shanghai Jiao Tong University Affiliated Sixth People's Hospital, No. 600 Yishan Road, Shanghai 200233 (China).

Tel. +86-21-64369181, E-Mail: wangy63cn@126.com

#Qingwei Zhu , Xiaozheng Ling , and Yunlong Yang contributed equally to this work.

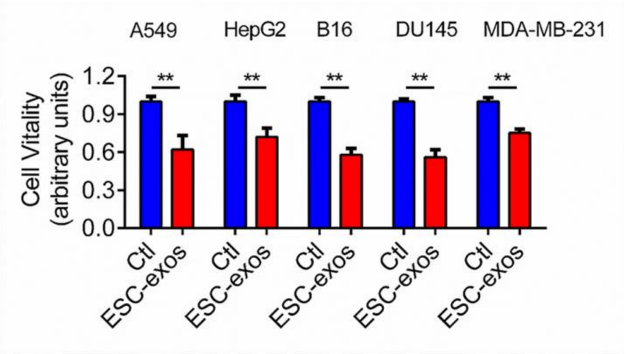

Supplement Figure 1. Anti-proliferative activity of ESC-exos in several cancer cells. lung cancer (A549), hepatoma (HepG2), melanoma (B16), breast cancer (MDA-MB-231) and prostate cancer (DU145) cells were treated with PBS (Ctl) or 1×10^9 particles/mL ESC-exos for 48h. The cell vitality was measured by the CCK-8 assay. Data represent average ± SD.

*p < 0.05 and **p < 0.01.

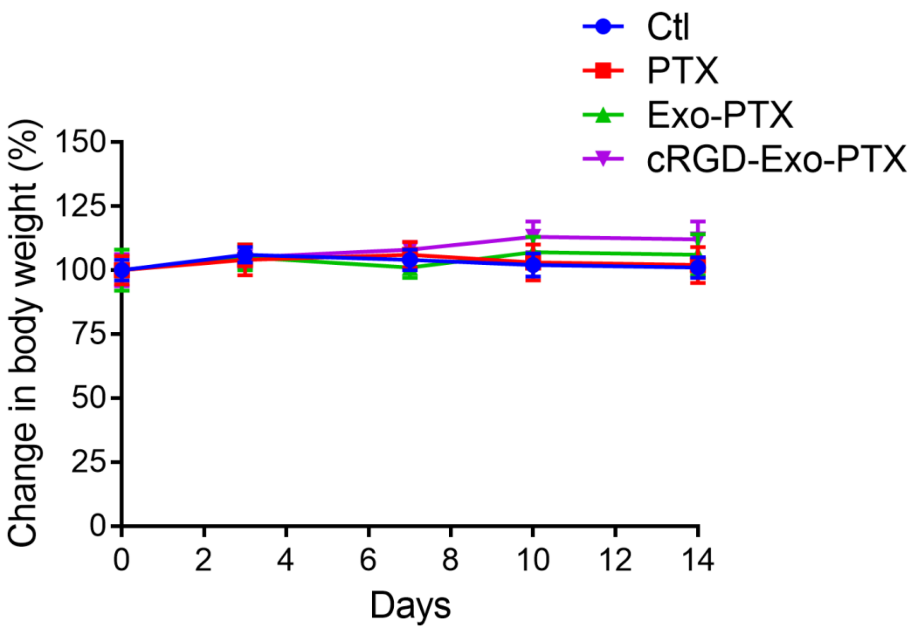

Supplement Figure 2. The body weight of subcutaneous xenograft mice in each group

The body weight of subcutaneous xenograft mice was measured in each group at day 0, 3, 7, 10, and 14 after treatment with PBS, PTX, Exo-PTX, and cRGD-Exo-PTX.
